# Cerebral ultrasound abnormalities in preterm infants caused by late-onset sepsis

**DOI:** 10.1371/journal.pone.0173227

**Published:** 2017-03-16

**Authors:** L. C. Claessens, I. A. Zonnenberg, F. A. M. van den Dungen, R. J. Vermeulen, M. M. van Weissenbruch

**Affiliations:** 1 Department of Neonatology, VU University Medical Center, Amsterdam, The Netherlands; 2 Department of Child Neurology, Neuroscience Campus Amsterdam, VU University Medical Center, Amsterdam, The Netherlands; Centre Hospitalier Universitaire Vaudois, FRANCE

## Abstract

**Introduction:**

This study describes cerebral ultrasound abnormalities caused by late-onset sepsis (LOS) in very preterm infants with a gestational age of < 32 weeks and/or birthweight < 1500 grams.

**Methods:**

The prospective study (“INFANT study”) included 117 preterm infants with suspected LOS. Proven LOS was defined as a positive blood culture after 72 hours of life. In case of coagulase-negative staphylococci an elevated C-reactive protein was additionally required to establish proven LOS. Patients were identified as proven LOS and patients with only clinical symptoms of LOS. Cerebral ultrasound images were obtained in the first week after birth, during/after LOS and before discharge. Cerebral findings were divided in no/minor and major abnormalities.

**Results:**

Eighty-six preterm infants had proven LOS and 31 preterm infants had only clinical signs of LOS. Four infants were excluded because pre-existing major brain abnormalities. No significant differences (p = 0.624) for incidence of major brain abnormalities on cerebral ultrasound were found.

**Conclusion:**

No differences were revealed in prevalence of major brain abnormalities between the groups with proven LOS and with clinical signs of LOS. Both infants with a gram negative sepsis developed major brain abnormalities, whereas only two of 66 preterm infants coagulase-negative staphylococci sepsis developed major brain abnormalities.

## Introduction

Late-onset sepsis (LOS) has a large impact on the neurodevelopment of very preterm infants (gestational age < 32 weeks) and/or very low birth weight infants (birth weight < 1500 gram) characterized by an increased risk for developing cognitive impairments, cerebral palsy and other neurodevelopmental disabilities compared to preterm infants without a sepsis.[1–5] Earlier studies have shown that up to 40% of the very preterm infants (born < 32 weeks gestational age) had at least one episode of LOS during their Neonatal Intensive Care Unit (NICU) stay.[6–9] Moreover, infants with a birth weight < 1000 grams were more likely to develop an infection.[9] Overall, half of the preterm infants with clinical symptoms of LOS showed a positive blood culture, in most cases coagulase-negative staphylococci (CoNS).[2;7;10]

Several studies have revealed that preterm infants with a proven infection were more likely to develop periventricular leukomalacia (PVL).[1;3] PVL grade II and III (cystic form), associated with the worst neurodevelopmental outcome, occurs in up to 1.3% of the preterm infants and is a strong predictor of cerebral palsy.[1;11] A more common brain injury is the germinal/intra ventricular hemorrhage (IVH) that affects 30% of the preterm infants.[12] IVH can be complicated by post-hemorrhagic ventricular dilation or periventricular venous infarction. These complications are related to cerebral palsy and cognitive impairment.[13;14]

Transient abnormalities and changes of the preterm brain during an infection episode have not been fully described yet. Moreover, it is important to know how different bacterial agents causing LOS may act on the developing preterm brain. Short-term abnormalities can be diagnosed using ultrasonography or MRI. MRI as a neuroimaging technique is preferred because of its high sensitivity and specificity, but has also disadvantages for a preterm infant. MRI cannot be performed bedside and the use of sedatives is frequently necessary. Ultrasound as a neuroimaging technique, however, has the advantage it can easily be performed longitudinally and bedside.

The aim of this study was to investigate the abnormalities seen on cerebral ultrasound during and after LOS.

## Methods

### Study population

Preterm infants with a gestational age of < 32 weeks and/or birth weight < 1500 gram admitted to the level III NICU of the VU University Medical Center between March 2008 and December 2014. Parents were asked for informed consent to participate in the “INFANT” study, a prospective study investigating immunogenetic, pharmacological and neurodevelopmental aspects of LOS and meningitis in preterm infants. All preterm infants with a suspected LOS were prospectively included. Infants with syndromal or chromosomal abnormalities and congenital metabolic disorders were excluded. Informed parental written consent was obtained and approval was given by the medical ethical committee of the VU University Medical Center. An observational study was performed, due to a lack of information on the outcome parameter in this age group it was not possible to perform a power calculation to calculate sample size.

### Late-onset sepsis

LOS was suspected when one of the following clinical symptoms occurred: hypothermia (<36,5°C) or hyperthermia (>37,5°C) or temperature instability, hypotension, tachycardia, apnea, feeding problems, irritability and/or apathy. Proven LOS was defined as a positive blood culture (BACTEC Peds Plus^™^/F Medium; Becton Dickinson) after 72 hours of life. During analysis, if PCR was available for research purposes, it was used to differentiate between causal agents. In case of coagulase-negative staphylococci an elevated C-reactive protein within 2 days of blood culture was additionally required for a final diagnosis. LOS was considered proven if blood culture and/ or PCR turned positive. When blood culture did not turn positive but the clinical signs implied antibiotic treatment for seven days, LOS was considered probable but not proven.[4;9]

All preterm infants who were suspected for LOS received empirical treatment with intravenously administered amikacin and penicillin G for at least 2 days. Depending on the causal agent found by blood culture, this combination of antibiotics was continued or was switched to another antibiotic regime. Antibiotics were discontinued, when blood culture was sterile and CRP was not elevated. However, in case of clinical LOS or elevated CRP, empirical antibiotics were continued for seven days.

### Cerebral ultrasound

Cerebral ultrasound images, which were performed as standard of care, were retrospectively collected from the medical chart of the preterm infant and the database of the Radiology Department and analyzed. Cerebral ultrasound was performed using a real-time scanner (AlokaProsound α7, Aloka co., Ltd., Tokyo, Japan). Images were obtained using a 7,5 MHz transducer. Images were recorded in at least five coronal and five sagittal planes using the anterior fontanel as an acoustic window. In addition, the mastoid fontanel was used as an acoustic window for observing the midbrain, posterior fossa and the ventricular system. Preterm infants with major brain abnormalities seen on ultrasound in the first week after birth were excluded from analysis.

During their stay in the NICU, all preterm infants underwent at least three cerebral ultrasounds. One cerebral ultrasound was performed in the first week after birth by a pediatric radiologist. The attending neonatologist performed sequential cerebral ultrasounds. At least one cerebral ultrasound was performed during LOS. The last cerebral ultrasound was performed before discharge. To identify ultrasound abnormalities these findings had to be seen in at least two planes. These abnormalities were divided in no or mild abnormalities and major abnormalities.

**Fig 1:**
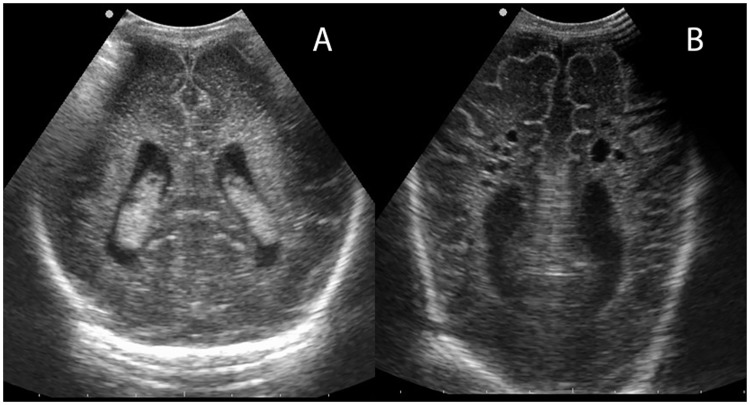
Minor and major brain abnormality on ultrasound images. A)Periventricular echodensities/mild PVL (grade 1) B) Cystic form of PVL.

Grading of the intraventricular hemorrhage (IVH) was according to Volpe[15] and periventricular leukomalacia (PVL) according to de Vries et al.[16] Mild abnormalities included IVH grade 1 (germinal matrix hemorrhage), grade 2 (10–50% of the lateral ventricle is filled with blood) and PVL grade 1 (transient echogenicity at least for seven days), inhomogeneous and/or seriously increased echogenicity, plexus cysts. Fig 1A shows an example of mild PVL/echodensities. Major abnormalities included abnormalities of the basal ganglia, IVH grade 3 (> 50% of the lateral ventricle is filled with blood, usually with distension of the ventricle), periventricular venous infarction, post-hemorrhagic ventricular dilation, PVL grade 2 (small fronto-parietal cystic lesions), grade 3 (extensive periventricular cystic lesions) and grade 4 (extensive cystic lesions in the deep white matter). Fig 1B shows an example of cystic PVL. Images were scored in consensus by IZ and LC.

### Clinical data

Clinical data were collected from the patients’ medical charts. Maternal variables included maternal pre-eclampsia/HELLP syndrome, chorioamnionitis, maternal group B streptococcal infection, usage of steroids and premature prolonged rupture of membranes. Neonatal variables included gestational age at birth, birth weight, gender, CRIB-score I (clinical risk index for babies), days of ventilation, > 24 hours steroid treatment for either respiratory or circulatory insufficiency, lumbar puncture for diagnostic reasons, hemodynamic significant patent ductus arteriosus (HS-PDA), surgery, necrotizing enterocolitis (NEC) stage 2 or 3 according to Bell[17], duration of admittance and death.

### Statistical analysis

Data were analyzed with SPSS using version 21 (SPSS Inc, Chicago, Illinois, USA). Statistical analyses were performed with Chi-square using Fisher’s exact test as appropriate for dichotomous data. A probability P value < 0.05 was considered statistically significant.

## Results

### Patient characteristics

During the study-period, 117 preterm infants (58 male, 59 female) were included. Eighty-six preterm infants were identified with a proven LOS and 31 preterm infants only had clinical symptoms of LOS, as described before. The most frequently observed symptoms were apnea, apathy and temperature instability. CoNS (n = 67) were the causal agents in 78% of LOS. *Staphylococcus aureus* (n = 12), *Escherichia coli* (n = 2), *Streptococcus agalactiae* (n = 1), *Klebsiella oxytoca* (n = 1), Gram positive rod (not further specified) (n = 1) and a mixture of CoNS and *Staphylococcus aureus* (n = 2) were the remaining causal agents. In total six preterm infants had a positive cerebrospinal fluid culture. In four infants *Staphylococcus aureus* (n = 3) or *Escherichia coli* (n = 1) was found, suggesting meningitis. In two CoNS were found, possibly due to contamination.

The characteristics of the maternal and neonatal data of both groups are shown in Table 1, showing only significant differences between type of infection groups in gender, confirming the overrepresentation of CoNS infection in the study group.

**Table 1:** Patient characteristics.

Clinical parameters	Proven LOS (n = 86)		Clinical LOS (n = 31)	p-value
	CNS (n = 67)	Others (n = 19)		
**Maternal characteristics**				
Maternal chorioamnionitis, *n(%)*	18 (27)	6 (32)	10 (32)	0.815
Pre-eclampsia/HELLP, *n(%)*	20(30)	7(37)	10(32)	0.855
GBS infection, *n(%)*	5 (7)	1 (5)	2 (6)	0.938
Antenatal steroids, *n(%)*	58 (87)	18 (95)	27 (87)	0.687
Premature prolonged rupture of membranes, *n(%)*	8 (12)	4 (21)	5 (16)	0.589
**Neonatal characteristics**				
Male gender, *n(%)*	37 (55)	6 (32)	15 (48)	<0.001
Female gender, *n(%)*	30 (45)	13 (68)	16 (52)	<0.001
Gestational age in weeks, *mean (range)*	28 (24–32)	27 (24–33)	27 (24–31)	0.769
Birth weight, *mean (range)*	1112 (545–1965)	960 (550–1800)	1011 (405–1855)	0.375
Lumbar puncture, *n(%)*	41 (61)	13 (68)	17 (55)	0.629
CRIB, *mean (range)*	3 (0–15)	4 (0–12)	5 (0–12)	0.484
Respiratory support (days), *mean (range)*	11 (0–87)	13 (0–44)	12 (0–51)	0.255
Steroid therapy > 24 hours, *n(%)*	10 (15)	3 (16)	4 (13)	0.979
NEC grade II/III, *n(%)*	11 (16)	5 (26)	4 (13)	0.462
Hemodynamic significant PDA, *n(%)*	17 (25)	5 (26)	11 (35)	0.574
Surgery, *n(%)*	4 (6)	3 (16)	2 (6)	0.350
Duration of admission (days), *mean (range)*	39 (10–116)	41 (6–105)	41 (8–91)	0.073
Died during admission, *n(%)*	5 (7)	4 (21)	3 (12)	0.350

Abbreviations used: LOS = late-onset sepsis; HELLP = Hemolysis Elevated Liver Enzymes and Low Platelets; GBS = group B Streptococcal; NEC = Necrotizing enterocolitis; PDA = patent ductus arteriosus; n = number

### Major abnormalities on cerebral ultrasound

In total, 409 cerebral ultrasounds (mean 3 per infant, range 2–5) were collected. Mild white matter abnormalities were seen in approximately one third of the patients, both in the proven and clinical LOS group.

The initial cerebral ultrasound (n = 114) performed in the first week of life showed major abnormalities in four out of 114 preterm infants. These four infants showed an intraventricular hemorrhage with venous infarction and were excluded for analysis. In none of these infants brain abnormalities worsened after an infection episode. Two of these four infants died due to redirection of care due to the combination of severe cerebral abnormalities and multiple organ failure following the infectious episode. In three infants no initial ultrasound was available. An overview of all brain abnormalities is shown in Table 2.

**Table 2:** Brain abnormalities seen on cerebral ultrasound during and/or after LOS*.

	Proven LOS (n = 84)		Clinical LOS (n = 29)
	CNS (n = 66)	Others (n = 18)	
IVH grade I (n)	6	4	2
IVH grade II (n)	5	1	1
IVH grade III (n)	1	1	1
IVH grade IV (n)	0	0	0
PHVD (n)	0	1	0
PVL grade I (n)	23	4	10
PVL grade II (n)	0	0	0
PVL grade III (n)	0	0	0
PVL grade IV (n)	0	0	1
Echodensities of thalamus/basal ganglia(n)	2	2	0

* Preterm infants with major brain abnormalities in the first week after birth were excluded.

Abbreviations used: LOS = late-onset sepsis; CNS = coagulase-negative staphylococci; IVH = intraventricular hemorrhage; PHVD = posthemorrhagic ventricular dilation; PVL = periventricular leukomalacia; n = number

Nine preterm infants, two with only clinical symptoms of LOS and seven with a proven LOS (six with positive blood culture and/ or PCR, one with positive PCR), developed major brain abnormalities during or following their infectious episode (Tables 3 and 4). Two of these infants developed major brain abnormalities two days prior to the suspected infectious episode.

**Table 3:** Major brain abnormalities seen on cerebral ultrasound during and/or after LOS*.

Case number	Infection	Major abnormalities*	PCR confirmed
# 17	*Staphylococcus aureus*	Infarction of the thalamus	Yes
		Intraventricular hemorrhage Grade II	
# 22	*Escherichia coli*	Intraventricular hemorrhage Grade III	Yes
		Posthemorrhagic ventricular dilatation	
*#33*	Coagulase-negative staphylococci	Echodensities thalamus	Yes
*#49*	*Streptococcus agalactiae*	Ventriculitis	Yes
		Widening of the ventricles	
*#50*	*Staphylococcus aureus*	Intraventricular hemorrhage Grade I	Yes
		Infarction of the thalamus	
*#72*	Coagulase-negative staphylococci	Echodensities of the basal ganglia	Yes
		Periventricular leukomalacia Grade I	
		Loss of white matter	
		Widening of the ventricles	
*#97*	Coagulase-negative staphylococci	Intraventricular hemorrhage Grade III	No PCR available

* Preterm infants with major brain abnormalities in the first week after birth were excluded.

**Table 4:** Major brain abnormalities seen on cerebral ultrasound during and/or after an episode of clinical symptoms of LOS, with a negative blood culture and negative PCR.

Case number	Major abnormalities*
# 26	Intra ventricular hemorrhage Grade III + IV
	Venous infarction
	Widening of the ventricles
# 35	Periventricular leukomalacia Grade IV
	Loss of white matter

* Preterm infants with major brain abnormalities in the first week after birth were excluded.

In the group of infants who developed major brain abnormalities during or following LOS (n = 7) blood culture revealed *Staphylococcus aureus* (n = 2), coagulase-negative staphylococci (n = 3) and *Escherichia coli* (n = 1). One preterm infant had both LOS caused by *Streptococcus agalactiae* and meningitis with *Escherichia coli* as causal agent.

Both patients with LOS caused by *Staphylococcus aureus* (n = 2) showed echo densities of the thalamus, which was an indication of a thalamus infarction. This was confirmed with MRI, which was performed at 40 weeks gestational age of the preterm infant. In both patients had iv catheters, but no thrombi on the tip or nearby the tip were found when investigated by ultrasound.

In addition, the infants with a proven coagulase-negative staphylococci LOS (n = 3) showed in one infant an intraventricular hemorrhage grade III and in two infants echo densities of the basal ganglia. In all these infants the abnormalities were observed after onset of the infectious episode. One of them developed a periventricular leukomalacia and loss of white matter tissue and eventually died due to therapy resistant respiratory insufficiency. In the other infant, the echo densities of the basal ganglia, seen during the infectious episode on ultrasound, were no longer visible on MRI at term age. Escherichia coli was isolated from cerebrospinal fluid indicating meningitis in combination with LOS caused by *Streptococcus agalactiae* in one patient. Another infant with proven LOS with *Escherichia coli* meningitis was suspected, however cerebrospinal fluid remained sterile. On ultrasound both infants presented the image of ventriculitis and ventricular hemorrhage resulting in dilatation of the ventricles.

In the group with only clinical symptoms of LOS (n = 31), two preterm infants developed major brain abnormalities on cerebral ultrasound; one infant developed intraventricular hemorrhage bilaterally with venous infarction and ventricle dilatation, that occurred two days prior to the presentation of clinical symptoms of infection; one infant developed cystic PVL four weeks after the infectious episode (Table 3)

Overall, during and/or after an infection episode no significant differences (p = 0.624) were found in incidence of major abnormalities detected on cerebral ultrasound between the group of preterm infants with a proven LOS and the group with a probable LOS (clinical symptoms but negative blood culture).

## Discussion

This study shows that during a sepsis episode there is no difference in major brain ultrasound abnormalities between infants with a proven LOS and infants with only clinical symptoms of LOS. In two infants abnormalities were found prior to the sepsis episode, which might suggest other mechanisms might be of importance. Mild white matter abnormalities are present in one third of all patients.

The overall findings of the current study are not in line with earlier studies, indicating that culture positive sepsis is an important risk factor for ventricular dilation, periventricular leukomalacia (PVL) and intraventricular hemorrhage (IVH) in preterm infants.[3;4;18–20] A possible explanation might be that in our study coagulase-negative staphylococci were responsible for 78% of the proven LOS. Only three preterm infants developed a proven LOS caused by gram negative bacteria and one preterm infant a proven meningitis caused by *Escherichia coli*. In our study population, LOS caused by coagulase-negative staphylococci was not associated with more echo abnormalities in general than other causal agents or clinical infection episodes.

Earlier studies have shown that gram-negative bacteria cause the highest mortality among preterm infants.[9;21] Our findings support these results; two out of four preterm infants who developed an infection caused by gram-negative bacteria died during the infection episode. The current study also showed that an infection with *Escherichia coli* has the largest impact on the developing brain; two out of three preterm infants developed major brain abnormalities (e.g. ventriculitis and ventricular hemorrhage grade III), which in both cases resulted in dilation of the ventricles. These findings suggest that infection with gram-negative bacteria not only have a large impact on the mortality rates, but may also cause short-term changes of the developing preterm brain. Therefore, cerebral MRI after a proven LOS has to be considered.

An unanticipated finding in our study was that 14% (n = 2) of the preterm infants infected with *Staphyloccocus aureus*, developed abnormalities of the thalamus without other major brain abnormalities on cerebral ultrasound.

Hemodynamically significant patent ductus arteriosus (HS-PDA) alone is a known risk factor for major brain abnormalities.[22–24] In the present study we observed that preterm infants with a HS-PDA were more prevalent in the group with only clinical symptoms of LOS. Possibly, a HS-PDA mimics the clinical symptoms of LOS, leading to an overestimation of the number of preterm infants with probable LOS.

Strengths of our study include its prospective element. Because of the prospective design of this study, PCR could be used to differentiate between causal agents found by blood culture. Blood culture is the gold standard for diagnosis of LOS in preterm infants, but adding PCR increases the specificity of bacteria causing LOS by informing us about the specific bacterial load.[25;26] Furthermore, long term follow up will be achieved for all included preterm infants over time which will provide more information about the neurodevelopmental outcome of preterm infants with minor and major brain abnormalities seen on cerebral ultrasound during a sepsis episode.

We also acknowledge several limitations of our study. This is a single center study with a relatively small number of preterm infants that were included in this study. Due to our strict criteria for suspicion of LOS and thus inclusion in our study, most of the preterm infants had a positive blood culture. As a result the reference group without positive blood culture is limited. This, and the rare occurrence of adverse outcome, does not allow statistical modelling. Furthermore, the high incidence of coagulase-negative staphylococci limits the number of other infections in this study group, leading to a small group of patients with a proven LOS caused by other bacteria. Therefore, it is difficult to assess the prevalence of major brain abnormalities in LOS caused by other bacteria than coagulase-negative staphylococci. Cerebral ultrasound images were analyzed. Not all preterm infants in our study had been subjected to three cerebral ultrasounds (13%). Further studies are needed before the association between LOS and the development of brain abnormalities can be more clearly understood. In fact, to minimize missing data cerebral ultrasound images should have been prospectively gathered the day at onset (day 0), day 3 and day 7 after the onset of (probable) LOS.

In conclusion, this study cerebral ultrasound imaging has shown that only two of the 66 preterm infants with a proven LOS caused by coagulase-negative staphylococci developed major cerebral abnormalities. These findings might suggest a minor role in at least short-term term effects of coagulase-negative staphylococci in the developing preterm brain. For now follow-up of these preterm infants, has to specify if the short-term outcome of coagulase-negative staphylococci is a good predictor in the neurodevelopmental outcome further in life.

## Abbreviations

CoNS: Coagulase-negative Staphylococci
CRIB: Clinical Risk Index for Babies
CRP: C-Reactive Protein
HELLP: Hemolysis Elevated Liver Enzymes and Low Platelets
HS-PDA: Hemodynamic Significant Patent Ductus Arteriosus
IVH: Germinal/Intra Ventricular Hemorrhage
LOS: Late-Onset Sepsis
MRI: Magnetic Resonance Imaging
NEC: Necrotizing Enterocolitis
NICU: Neonatal Intensive Care Unit
PCR: Polymerase Chain Reaction
PVL: Periventricular Leukomalacia
